# Supplementary material for: Visual feature analysis on selective appetite in individuals with autism spectrum disorders
Source: PLoS One. 2025 Jun 6;20(6):e0325416. doi: 10.1371/journal.pone.0325416 (PMC12143564; doi:10.1371/journal.pone.0325416)
Supplement: S1 Appendix — (DOCX) [file pone.0325416.s001.docx]

**Appendix 1. Cumulative contribution ratio of principal contribution analysis (PCA) for the list of interview questions**

　The contributions of each principal component are listed in Table S1. A cumulative contribution ratio of 0.8 is commonly used as a standard criterion. Therefore, this study adopted a threshold of 0.8.
