# Supplementary material for: Visual feature analysis on selective appetite in individuals with autism spectrum disorders
Source: PLoS One. 2025 Jun 6;20(6):e0325416. doi: 10.1371/journal.pone.0325416 (PMC12143564; doi:10.1371/journal.pone.0325416)
Supplement: S2 Appendix — (DOCX) [file pone.0325416.s002.docx]

**Appendix 2. Comparison of the explanatory power between Non-negative Matrix Factorization (NMF) and Principal Component Analysis (PCA)**

An investigation was conducted for both autism spectrum disorders (ASD) and typical development (TD) groups to determine whether NMF or PCA provided higher explanatory power. Specifically, the following four logistic regressions were conducted with adjusted *R²* as the accuracy indicator:

- The 11-dimensional vectors extracted by *NMF* were used as explanatory variables and the average preference ratings of the *ASD* group were used as response variables.
- The 11-dimensional vectors extracted by *NMF* were used as explanatory variables and the average preference ratings of the *TD* group were used as response variables.
- The 11-dimensional vectors extracted by *PCA* were used as explanatory variables, and the average preference ratings of the *ASD* group were used as response variables.
- The 11-dimensional vectors extracted by *PCA* were used as explanatory variables, and the average preference ratings of the *TD* group were used as response variables.

The results of this analysis are listed in Table S1. NMF demonstrated higher explanatory power than PCA in both the ASD and TD groups.

ASD, autism spectrum disorder; TD, typical development; NMF, non-negative matrix factorization; PCA, principal contribution analysis
