# Supplementary material for: Visual feature analysis on selective appetite in individuals with autism spectrum disorders
Source: PLoS One. 2025 Jun 6;20(6):e0325416. doi: 10.1371/journal.pone.0325416 (PMC12143564; doi:10.1371/journal.pone.0325416)
Supplement: S1 Table — (DOCX) [file pone.0325416.s003.docx]

**Table S1.** Contributions of each principal component

| Principal component | Contribution ratio |
| --- | --- |
| 1 | 0.224 |
| 2 | 0.153 |
| 3 | 0.124 |
| 4 | 0.079 |
| 5 | 0.052 |
| 6 | 0.042 |
| 7 | 0.035 |
| 8 | 0.029 |
| 9 | 0.025 |
| 10 | 0.019 |
| 11 | 0.016 |
| Cumulative contribution ratio | 0.801 |
