# Supplementary material for: Visual feature analysis on selective appetite in individuals with autism spectrum disorders
Source: PLoS One. 2025 Jun 6;20(6):e0325416. doi: 10.1371/journal.pone.0325416 (PMC12143564; doi:10.1371/journal.pone.0325416)
Supplement: S2 Table — (DOCX) [file pone.0325416.s004.docx]

**Table S2.** Adjusted *R*^2^ for regression analysis

|  | NMF | PCA |
| --- | --- | --- |
| ASD | 0.038 | 0.036 |
| TD | 0.065 | 0.058 |
