# Supplementary material for: Visual feature analysis on selective appetite in individuals with autism spectrum disorders
Source: PLoS One. 2025 Jun 6;20(6):e0325416. doi: 10.1371/journal.pone.0325416 (PMC12143564; doi:10.1371/journal.pone.0325416)
Supplement: S1 File — (PDF) [file pone.0325416.s007.pdf]

（第１３条関係）
受付 2021-C014

2021 年 9 月 27 日

医 学 研 究 等 倫 理 審 査 結 果 通 知 書

研究責任者
　寺田 和憲　殿

岐阜大学大学院医学系研究科医学研究等倫理審査委員会委員長
塚田　敬義

　申請のあった研究について、審査結果を下記のとおり通知します。

記

研究課題名	人が対象物の概念を知覚する際に用いる視覚的特徴の同定
審査区分	□　委員会審査（審査日：　年　月　日）<br>■　迅速審査（審査日：2021 年 9 月 27 日）
審査結果	■　承認<br>□　不承認<br>□　継続審査<br>□　停止<br>□　中止<br>□　非該当
「承認」以外の場合の理由等	
備考	

（第１４条第３項関係）
受付 2021-C014

2021 年 9 月 27 日

医 学 研 究 等 実 施 許 可 通 知 書

研究責任者
寺田 和憲　殿

岐阜大学大学院医学系研究科長　　中島　　茂
岐阜大学医学部附属病院長　　　　吉田　和弘

申請のあった研究について、下記のとおり決定したので通知します。

記

許可番号	27-230
研究課題名	人が対象物の概念を知覚する際に用いる視覚的特徴の同定
実施許可の判定	■　許可<br>□　不許可
備考	
