# Supplementary material for: Visual feature analysis on selective appetite in individuals with autism spectrum disorders
Source: PLoS One. 2025 Jun 6;20(6):e0325416. doi: 10.1371/journal.pone.0325416 (PMC12143564; doi:10.1371/journal.pone.0325416)
Supplement: S2 File — (PDF) [file pone.0325416.s008.pdf]

(Re: Article 13)

Reception 2021-C014

September 27, 2021

Ethical Review of Medical Research Notification of Results

principal investigator

Kazunori Terada Sir.

Chairman, Ethical Review Committee for Medical Research, Gifu University Graduate School of Medicine

Tsukada

Takayoshi

The results of the review of the research applications will be notified as follows.

record

subject for study (inquiry, enquiry)	Identification of visual features that people use to perceive the concept of an object
Examination Classification	☐ Committee Review (Date of Review: Year Date of Examination Date)<br>■ Expedited Review (Review date: September 27, 2021)
Results of Examination	■ approval<br>☐ disapproval<br>☐ carrying over a bill from one Diet session to the next, being deliberated by a committee in the interim<br>☐ suspension of music, dance, etc. as a sign of mourning<br>☐ suspension<br>☐ not applicable
Other than "Approval" Reason etc.	
remarks	

(Re: Article 14, Section 3)
Reception 2021-C014

September 27, 2021

Notification of Permission to Conduct Medical Research, etc.

principal investigator

Kazunori Terada Sir.

Dean, Graduate School of Medicine, Gifu University
Nakajima
Shigeru Nakajima, Director, Gifu University Hospital
Kazuhiro Yoshida Kazuhiro

We hereby notify you that we have made the following decisions on the research for which you have applied.

record

license number	27-230
subject for study (inquiry, enquiry)	Identification of visual features that people use to perceive the concept of an object
Determination of permission for implementation	■ permit<br>□ not permitting
remarks	
