# Supplementary material for: Visual feature analysis on selective appetite in individuals with autism spectrum disorders
Source: PLoS One. 2025 Jun 6;20(6):e0325416. doi: 10.1371/journal.pone.0325416 (PMC12143564; doi:10.1371/journal.pone.0325416)
Supplement: S3 File — (PDF) [file pone.0325416.s009.pdf]

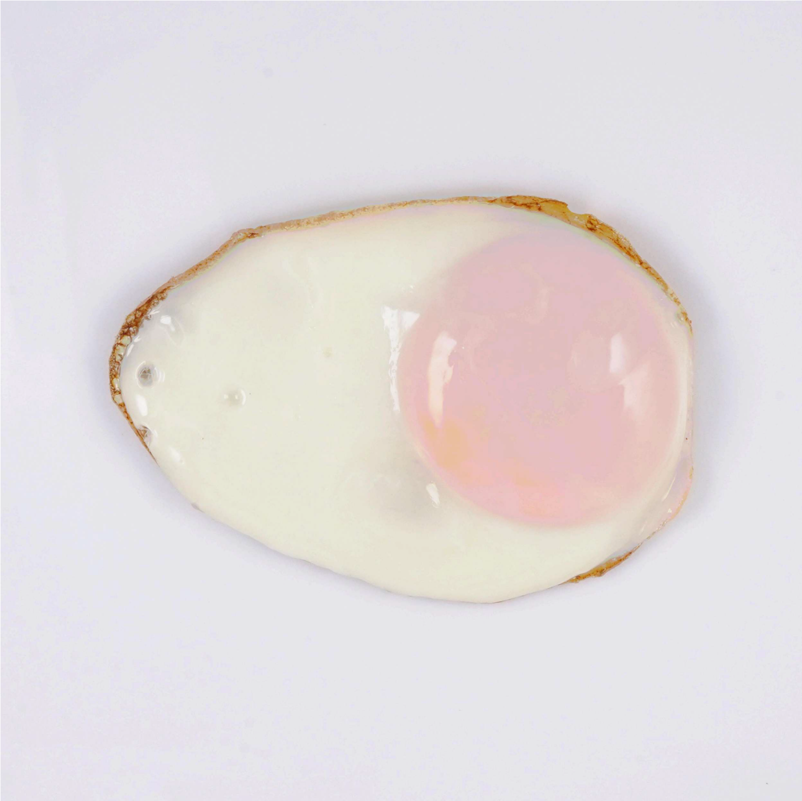

1/50枚目

１．この目玉焼きの**食べたさ**の程度を0から100までの数値でご回答ください．

全く食べたくない					どちらでもない					とても食べたい
0	10	20	30	40	50	60	70	80	90	100

２．どのような理由（観点）で「食べたい／食べたくない」と感じますか？できるだけ詳細にご記入ください．

※どんなに些細なことでも結構ですのでご記入ください．ただし，一度他の画像に対して記入していただいた理由（観点）は再度記入していただく必要はありません（していただいても結構です）．**新しい観点が出てきた場合のみご記入いただければ結構です**．

→
