# Supplementary material for: Visual feature analysis on selective appetite in individuals with autism spectrum disorders
Source: PLoS One. 2025 Jun 6;20(6):e0325416. doi: 10.1371/journal.pone.0325416 (PMC12143564; doi:10.1371/journal.pone.0325416)
Supplement: S4 File — (PDF) [file pone.0325416.s010.pdf]

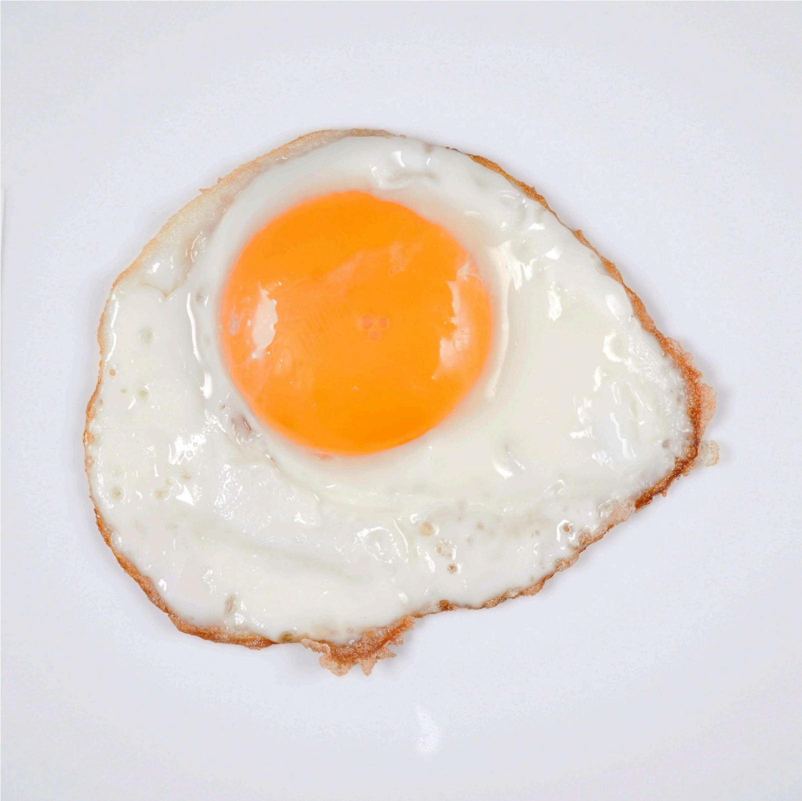

1/50

1. Please rate your **desire to eat** this sunny-side up egg on a scale from 0 to 100.

Not at all want to eat					Neither					Very much want to eat
0	10	20	30	40	50	60	70	80	90	100

2. What reasons (aspects) make you feel that you "want to eat / don't want to eat" this? Please describe in as much detail as possible.

Note: Any reason, no matter how minor, is welcome. However, you don't need to repeat reasons (aspects) that you've already mentioned for other images (though you may do so if you wish). **You only need to mention new aspects that you haven't mentioned before.**

→
