# Supplementary material for: Visual feature analysis on selective appetite in individuals with autism spectrum disorders
Source: PLoS One. 2025 Jun 6;20(6):e0325416. doi: 10.1371/journal.pone.0325416 (PMC12143564; doi:10.1371/journal.pone.0325416)
Supplement: Attachment — Submitted filename: Response to Reviewers.docx [file pone.0325416.s013.docx]

Reviewer #1: It’s my pleasure to review the manuscript

Good work.

Well written manuscript, clear abstract, good discussion and good work

Aim is well explained and Title is good, grammatically is good no must

Language good

Thank you for commending our study.

Reviewer #2: The authors conducted a Visual Feature Analysis (VFA study of how individuals (18-30 yrs) com Autism Spectrum Disorder (ASD) and Typical Development (TD) perceive and respond to visual stimuli, using a test based on 50 images of sunny-side-up eggs. In the context of Selective Appetite in Individuals with Autism Spectrum Disorder (ASD). The manuscript is clear and well-written; the authors thoroughly describe all study steps, including subject selection, groupcriteria, image development, test procedures, and statistical analysis.

The images, figures, and tables are well-presented.

Thank you for commending our study.

My only concern are:

- How number of subjects were defined?

Thank you for pointing this out. In this study, we conducted an a priori power analysis using G*Power within a linear regression framework, assuming a medium effect size (f² = 0.15), an alpha level of .05, and a power of .80. This analysis indicated that approximately 77 participants (i.e., about 39 participants per group) would be required. Considering potential missing data or insincere responses into account, we aimed to recruit 50 participants per group. Therefore, we have added the two following texts in Participants part of Materials and methods section.

**“We conducted an a priori power analysis using G*Power within a linear regression framework, assuming a medium effect size (f² = 0.15), an alpha level of .05, and a power of .80. This analysis indicated that approximately 77 participants (i.e., about 39 participants per group) would be required. Considering potential missing data or insincere response into account, we aimed to recruit 50 participants per group.”**

- Although the test does not have a direct application in practice, how do the authors suggest that it could evolve into a practical application?

As a practical application, we aim to provide caregivers of individuals with ASD with information on food preferences so that they can offer appropriate meals. We will also evaluate the effectiveness of this intervention in supporting healthier eating habits. Therefore, we have added the following text in the Conclusion section.

**“As a practical application, we aim to provide caregivers of individuals with ASD with information on food preferences so that they can offer appropriate meals. We will also evaluate the effectiveness of this intervention in supporting healthier eating habits.”**
